# The role of medium-frequency pulsed electrotherapy in patients with postoperative ileus after gynecological surgery

**DOI:** 10.3389/fsurg.2025.1699446

**Published:** 2026-01-19

**Authors:** Jiazhen Ji, Haixia Wu, Xiaopei Huang, Shouhua Yang

**Affiliations:** 1 Department of Gynecology, South China Hospital, Medical School, Shenzhen University, Shenzhen, Guangdong, China; 2 Department of Gynecology, Maternal and Child Health Hospital of Hubei Province, Wuhan, Hubei, China

**Keywords:** gynecology, medium-frequency pulsed electrotherapy, postoperative ileus, rehabilitation physiotherapy, retrospective analysis

## Abstract

**Objective:**

To investigate whether medium-frequency pulse electrical therapy can effectively shorten the duration of postoperative ileus in gynecological patients.

**Methods:**

Clinical data from 126 eligible gynecological surgery patients from January 2024 to June 2024 were analyzed. Patients were grouped based on whether they received medium-frequency pulse electrical therapy and the initiation time of the therapy. Postoperative time to first flatus was compared between groups.

**Results:**

There was no statistically significant difference in the time to first anal exhaust between patients who received medium-frequency pulsed electrotherapy and those who did not (*P* = 0.36). Similarly, no significant difference was observed based on the timing of initiation of medium-frequency pulsed electrotherapy (*P* = 0.70).

**Conclusion:**

Medium-frequency pulse electrical therapy shows no significant effect in shortening the duration of postoperative ileus in gynecological patients.

## Introduction

Postoperative ileus (POI), also known as postoperative intestinal paralysis, is defined as a transient interruption of normal bowel activity following abdominal or non-abdominal surgery due to various causes (1). Its duration and symptom severity vary, but it is an almost inevitable process for most patients after abdominal surgery (2). It differs from “paralytic ileus” in surgery, which is caused by inflammation, infection, etc., leading to weakened or absent intestinal peristalsis. Patients present with more severe abdominal pain, distension, possibly accompanied by nausea and vomiting, diminished or absent bowel sounds on auscultation, and dilated, gas- and fluid-filled intestines on abdominal x-ray or CT. Paralytic ileus and POI share similar pathophysiological processes, but their clinical manifestations and management decisions differ significantly. With continuous improvements in gynecological surgeons’ skills and the demand for minimally invasive and precise surgery, the primary approach in gynecological surgery has gradually shifted from open procedures to laparoscopic surgery. In China, statistics indicate that over 90% of female patients with benign or malignant diseases undergo laparoscopic surgery (3). For gynecological surgeries, minimally invasive techniques (including abdominal, vaginal, robotic, etc.) are associated with shorter hospital stays and faster recovery of bowel function and daily activities compared to traditional open surgery. Combining these techniques with the popular Enhanced Recovery After Surgery (ERAS) strategy can further shorten postoperative recovery time (4, 5). Antonio Simone Lagana et al. pointed out that the postoperative recovery process for patients with malignant tumors is significantly longer than for those with benign diseases. They believe that correct postoperative management and appropriate nursing care play important roles in improving treatment success rates and reducing complication rates (6). Both within ERAS theory and in various studies, the recovery of postoperative gastrointestinal function plays a crucial role (3). The duration of POI significantly impacts hospital stay, nursing costs, and the incidence of postoperative complications (such as pneumonia, poor wound healing). However, effective prevention and treatment methods for this condition are still lacking (7, 8).

Medium-frequency pulse electrical therapy is a novel rehabilitation physiotherapy modality. It delivers electrical stimulation at specific frequencies through a dedicated device to peripheral skin receptors, exciting local nerves, enhancing nerve conduction, and promoting local blood circulation, thereby accelerating tissue repair and enhancing body tolerance. Currently, domestic and international scholars have applied pulse electrical therapy in areas such as alleviating post-anesthesia pain (9), treating diabetic peripheral neuropathy (10), and postpartum pelvic floor rehabilitation (11). Results indicate positive effects of this therapy on body recovery, suggesting its value for application and promotion. However, there is limited research on the role of medium-frequency pulse electrical therapy in accelerating postoperative recovery in gynecology.

## Objective

To observe the effect of medium-frequency pulse electrical therapy on POI in gynecological surgery patients.

## Materials and methods

Patients: This study was a single-center retrospective cohort study. Hospitalized patients in the Department of Gynecology at South China Hospital, Shenzhen University, between January 28, 2024, and June 08, 2024 were selected. Inclusion and exclusion criteria are as follows:

Inclusion Criteria: Patients who underwent various gynecological internal genital surgeries in our department during the aforementioned period, including adnexal surgery, uterine corpus surgery, hysterectomy, via laparoscopic or open approaches, and under general anesthesia with endotracheal intubation.

Exclusion Criteria:

- Patients uncooperative with relevant treatments and/or with communication barriers;
- Patients with incomplete clinical statistical data.
- Patients undergoing intestinal resection during surgery.
- Patients with a history of mental illness, alcoholism, or drug abuse.
- Patients with electrical stimulation devices (pacemakers or implantable defibrillators).
- Patients participating in other clinical studies.
- Patients receiving epidural anesthesia.

Instruments: The instrument for medium-frequency pulse electrical therapy was: Medium-frequency Therapy Apparatus (YK-2000B, Guangzhou Yikang Medical Equipment Industrial Co., Ltd.). Application method: The patient assumed a supine position, with 2 electrode pads placed on the upper and lower abdomen each (avoiding the surgical incision). After connecting to the machine, the mode was set to “Abdominal Physiotherapy”, intensity at 8–10 levels. Each stimulation session lasted 30 min, performed twice daily until the day before discharge. Relevant parameters of the instrument: Operating voltage: 220 V, 50 Hz; Rated power: 50VA; Output frequency: 1k–10k Hz; Output waveform: Bidirectional square wave. The electrodes used were non-polarized. Blood potassium analysis: Vitros-5600 analyzer, used according to manufacturer protocols. Complete blood count: Mindray CS-7500 analyzer, used according to standard procedures.

Observation Indicator: Referring to the Guangdong Provincial Local Standard “DB44/T 1581-2015 Perioperative Postoperative Gastrointestinal Motility Evaluation Specification”, and considering the accuracy of data sources in retrospective analysis, this study used the objective and accurately recorded indicator of the time from surgery end to the first postoperative flatus as the observation endpoint. This retrospective study was approved by the Ethics Committee of South China Hospital, Shenzhen University (Approval No.: HNLS20240709003-A; date of approval 26/7/2024).

Statistical Analysis Methods: Data conforming to normal distribution are expressed as mean ± standard deviation, non-normally distributed data as median (interquartile range). Quantitative data were analyzed using variance tests and linear regression analysis; comparisons between grouped data were performed using non-parametric tests. All statistical analysis results with *P* < 0.05 were considered statistically significant. Data analysis was performed using IBM SPSS Statistics 25.0.

## Results

1. Patient characteristics: Patient baseline characteristics: All enrolled patients were female Asians, with ages ranging from 11 to 74 years, mean 38.86 ± 10.90 years.
2. Separate statistical analyses were performed based on whether medium-frequency pulse electrical therapy was used and the timing of its use. The results are shown in Table 1.
3. After obtaining the above results, finding no significant value of medium-frequency pulse electrical therapy in postoperative gastrointestinal function recovery, we proceeded with correlation analysis of influencing factors. The results are shown in Table 2.
4. Nonlinear regression was used to analyze the relationship between time to flatus and age, as well as operation duration. The summarized results are shown in Table 3.

**Table 1:** Comparison of postoperative time to flatus based on medium-frequency pulse electrical therapy use and initiation time.

Group details	Postoperative time to flatus^a^ (h)					H	*P*
The timing of medium-frequency pulsed electrotherapy initiation	Within 12 h postoperatively	12–24 h postoperatively	24–48 h postoperatively	48 h postoperatively	Not used	2.18	0.70
	29.96 (20.54,42.44) *n* = 34	29.25 (19.63,42.66) *n* = 41	33.25 (20.92,55.83) *n* = 7	43.88 (17.47,70.56) *n* = 4	26.17 (16.25,41.00) *n* = 39		
Use of medium-frequency electrotherapy	Non-users		Users			0.86	0.36
	25.63 (16.91,40.70) *n* = 39		30.83 (20.92,43.23) *n* = 82				

a Since the data did not follow a normal distribution, it was presented as the median (interquartile range) in hours.

**Table 2:** Analysis of factors influencing postoperative time to flatus.

Group details	Postoperative time to flatus^a^ (h)				H	*P*
Preoperative BMI (kg/m^2^)	<18.5	18.5–25	24–28	>28	0.12	0.98
	29.42 (15.42,45.68) *n* = 11	27.88 (20.52,38.65) *n* = 64	29.96 (19.92,43.27) *n* = 42	25.70 (20.48,40.05) *n* = 9		
Preoperative hypokalemia	Yes		No		4.36	0.06
	26.00 (16.85,40.10) *n* = 36		34.39 (21.44,45.39) *n* = 90			
Postoperative hypokalemia	Yes		No		4.57	0.03
	24.96 (15.98,38.24) *n* = 62		34.17 (21.08,45.91) *n* = 64			
Preoperative anemia status	None 26.88 (18.77, 40.13)	Mild 30.83 (19.54, 43.92)		Moderate 37.42	2.60	0.45
	*n* = 94	*n* = 26		*n* = 1		
History of abdominal surgery	No		Yes		0.01	0.91
	27.67 (18.42,42.42) *n* = 71		20.41 (8.97,30.83) *n* = 55			
Surgical site	Adnexa	Uterine Corpus		Total Hysterectomy	15.53	<0.01
	24.18 (15.48,36.26) *n* = 68	33.92 (22.25,56.83) *n* = 23		34.67, (24.50,50.42) *n* = 35		
Surgical approach (3.1)	Single-port Laparoscopy	Multi-port Laparoscopy		Open	5.10	0.08
	27.88 (19.87,41.17) *n* = 82	27.50 (18.88,42.42) *n* = 39		70.47 (30.81,86.43) *n* = 5		
Surgical approach (3.2)	Laparoscopic		Open		5.03	0.03
	27.67 (19.38,41.23) *n* = 121		70.47 (30.81,86.43) *n* = 5			
Surgical approach (3.3)	Single-port Laparoscopy		Multi-port Laparoscopy		0.06	0.80
	20.88 (19.85,41.17) *n* = 82		27.50 (18.88,42.42) *n* = 39			

a Since the data did not follow normal distribution, it was presented as the median (interquartile range) in hours.

**Table 3:** Nonlinear regression analysis of factors influencing time to flatus.

Influencing factor	Number of cases	Correlation coefficient	*P*
Operation duration (h)	126	0.36	<0.01
Age (y)	126	0.10	0.26

## Discussion

The diagnosis and treatment of POI present certain difficulties in clinical practice. Firstly, there is still no consensus in the academic community on the definition of POI. Most literature suggests that symptoms related to POI should resolve spontaneously within 96 h. If patients cannot tolerate oral intake and bowel motility does not recover beyond this period, it is no longer defined as POI (12). Secondly, neglect of POI is also an important factor. Luke Traeger et al., in a meta-study, pointed out that the underestimation of POI incidence by clinicians leads to annual healthcare costs as high as 3.9 billion euros (8). In clinical practice, there is no consensus on effective assessment methods. Studies show that the recovery of the migrating motor complex does not equate to the recovery of normal function; meaning, the return of bowel sounds on auscultation does not fully represent the restoration of normal intestinal motility (13).

The etiology of POI remains unclear. Pathophysiological research suggests that at least the following mechanisms are involved in its development: 1. Effect of anesthetic drugs. Particularly the use of opioid analgesics, which activate opioid receptors on gastrointestinal smooth muscle, inhibiting gastric emptying and intestinal motility (2). 2. Sympathetic nerve excitation (14). Stimulated by pain, stress, and anesthetic drugs, the sympathetic nerves are excited, releasing norepinephrine from synaptic terminals, which inhibits gastrointestinal motility by stimulating α-2 adrenergic receptors on intestinal wall cells, among other effects (15). 3. Local inflammatory response. Mast cells, macrophages, etc., distributed in the intestinal wall, release various inflammatory mediators such as TNF-α, interleukin-1α, etc., under stimuli like mechanical manipulation, contributing to dysfunction of gastrointestinal smooth muscle cells (16). Additionally, preoperative fasting, intraoperative fluid overload causing intestinal edema, and postoperative electrolyte imbalances also participate in or contribute to this pathophysiological process (16). The interaction of these mechanisms causes gastrointestinal smooth muscle to lose its normal rhythmic and regular contractions, ultimately leading to POI.

Previous studies have shown that the recovery time of different parts of the digestive tract postoperatively exhibits segmental differences: the small intestine recovers within 0–24 h, the stomach within 24–48 h, and the colon within 48–72 h (17). In clinical work, when patients report spontaneous anal flatus or defecation, their abdominal distension symptoms can be effectively relieved. Spontaneous anal flatus is an essential part of the normalization of gastrointestinal motility after surgery. If gastrointestinal motility cannot restore its normal rhythm and coordination, the incidence of postoperative ileus, intestinal adhesions, etc., will significantly increase, which is detrimental to patient recovery and increases the average hospital stay and economic burden on the healthcare system. According to incomplete statistics, patients with POI have an approximately 4-day longer average hospital stay and an additional per capita medical cost of about $7,300 compared to the average (18). Shortening the duration of POI is also an important measure to reduce postoperative discomfort and decrease the average length of hospital stay.

Multiple studies and reports have found that commonly used methods to promote gastrointestinal motility postoperatively are controversial (1). These include sham feeding therapy (like chewing gum) (19), indwelling gastrointestinal drainage tubes, and medications (such as erythromycin, metoclopramide, non-steroidal anti-inflammatory drugs, etc.), which have not yielded satisfactory results in designed randomized controlled trials (2, 20). Among them, NSAIDs are a “double-edged sword”. By reducing the release of inflammatory factors and the impact of inflammatory mediators on intestinal function, they accelerate the recovery of bowel function; however, the weakening effect of these drugs on the mucosal barrier cannot be ignored. Postoperative patients, due to dietary restrictions, stress responses, and other factors, have compromised gastrointestinal mucosal barrier function. Perioperative use of these drugs is more likely to cause gastroduodenal ulcers, and in specific populations, increases the risk of intestinal perforation or anastomotic leakage (17). Alvimopan is a peripherally acting selective μ-opioid receptor antagonist. It can antagonize the negative effects of opioids on gastrointestinal smooth muscle cells without affecting their analgesic efficacy, promoting postoperative gastrointestinal motility and reducing the risk of ileus (21). However, due to its relatively high cost and increased risk of cardiovascular adverse events, its clinical use is somewhat limited.

In practice, a method that can effectively shorten POI duration without increasing the risk of other complications is very important. Basic medical research has found that among neuroelectrophysiological factors affecting intestinal function, the vagus nerve cannot be ignored; its dysfunction can exacerbate intestinal inflammation and weaken the intestinal barrier function. In animal experiments, electrical stimulation of the vagus nerve in endotoxin model mice led to the release of acetylcholine, which binds to nicotinic receptors on cells, modulating the body's inflammatory response (22, 23). Animal studies have found that whether stimulating the peripheral vagus nerve or activating central cholinergic neurons (24), both can reduce levels of TNF-α and IL-6 in the peritoneum, thereby inhibiting neutrophil aggregation and thus improving intestinal motility (25).

The mechanism of action of medium-frequency pulse electrical therapy on the body is not fully understood. It is speculated that it may affect target organ function by influencing autonomic nerve activity. In small-sample controlled clinical trials, medium-frequency pulse electrical therapy has shown a certain degree of analgesic effect (26) and has a positive effect on chronic constipation in both adults and children (27, 28). During postpartum breastfeeding, it can reduce the intensity of pain caused by uterine contractions (28). However, the above conclusions still lack confirmation from multicenter, randomized, large-scale studies.

In our designed comparison, all patients received relatively active postoperative enhanced recovery management (including postoperative fluid control, early active feeding, early ambulation, etc.). On this basis, whether comparing the group that received medium-frequency pulse therapy with the group that did not (*P* = 0.36), or grouping patients according to the initiation time of medium-frequency pulse electrical therapy (*P* = 0.70), and even though no adverse reactions were reported in any of the treated patients, no shortening effect of medium-frequency pulse electrical therapy on patient POI was observed among the groups. The possible reasons are speculated as follows: 1. In the experiment by The et al., direct stimulation of the animal's nerve was used, whereas transcutaneous medium-frequency pulse electrical stimulation might not effectively influence vagus nerve neurotransmitter release (25). 2. In the development of POI in gynecological patients, electrolyte imbalances and the effects of anesthetic drugs might be more important. In this study, we also observed that operation duration (*P* < 0.01), postoperative hypokalemia (*P* = 0.03), surgical site (*P* < 0.01), and surgical approach (laparoscopic vs. open) (*P* = 0.03) were factors that caused or exacerbated POI in patients. The specifics are as follows: The duration of surgery showed a positive correlation with the severity of POI (*P* < 0.01), indicating that longer surgeries were associated with slower postoperative recovery of bowel function. The occurrence of postoperative hypokalemia also significantly prolonged the duration of POI (*P* = 0.03), highlighting the important influence of electrolyte balance on the recovery of intestinal motility. Both the surgical site and extent were closely related to POI (*P* < 0.01), with more extensive procedures and greater tissue resection correlating with a greater impact on bowel function and correspondingly longer recovery times from POI. Furthermore, a significant difference was observed in the impact of different surgical approaches on POI (*P* = 0.03): compared to traditional open surgery, laparoscopy reduced interference with gastrointestinal function. The study by Ruth B. Hennebery et al. also indicated that preoperative systemic complications (such as diabetes, hypothyroidism, etc.), intraoperative massive blood loss (>1000 mL), preoperative use of smooth muscle inhibitors (such as magnesium sulfate), and postoperative infection can significantly prolong postoperative gastrointestinal function recovery time, even causing paralytic ileus (29).

Therefore, in practice, selecting the appropriate surgical approach to reduce operation duration, and paying attention to the prevention and treatment of electrolyte imbalances during and after surgery, especially avoiding hypokalemia, are effective measures to shorten POI.

## Limitations

This study is also a retrospective study of a small clinical sample, with limitations such as the inability to finely control various variables (e.g., anesthetic drug use, postoperative fluid infusion, etc.), inability to accurately obtain some subjective patient indicators, and lack of long-term follow-up data. Additionally, due to the small number of cases in some subgroups, the results might have some bias. However, the results of this experiment are consistent with conclusions from multiple centers, proving its reliability to a certain extent. In future experimental designs, we will build upon this study, adding more observation indicators and reducing the influence of confounding factors.

## Conclusion

Medium-frequency pulse electrical therapy shows no significant efficacy in shortening the duration of postoperative ileus in gynecological patients.

## Data Availability

The datasets presented in this study can be found in online repositories. The names of the repository/repositories and accession number(s) can be found below: China National Medical Research Registration and Documentation Information System. pid:249685 https://www.medicalresearch.org.cn/.
